# Les abcès froids pariétaux thoraciques chez les sujets immunocompétents

**DOI:** 10.11604/pamj.2015.20.161.5773

**Published:** 2015-02-20

**Authors:** Hanane Benjelloun, Sanaa Morad, Nahid Zaghba, Abdelaziz Bakhatar, Najiba Yassine, Abdelkrim Bahlaoui

**Affiliations:** 1 Service des Maladies Respiratoires, Hôpital Ibn Rochd, Casablanca, Maroc

**Keywords:** Abcès froid, paroi thoracique, tuberculose, immunocompétence, prise en charge, cold abscess, chest wall, tuberculosis, immunocompetent, management

## Abstract

Les abcès froids de la paroi thoracique représentent une forme rare et inhabituelle de tuberculose extrapulmonaire. Sa fréquence est estimée à moins de 5% des tuberculoses ostéoarticulaires, évaluées elles-mêmes à 15% des tuberculoses extrapulmonaires. L'objectif de ce travail est de rapporter la prise en charge diagnostique et thérapeutique de cette localisation dans notre structure. Etude rétrospective portant sur 18 cas colligés au service des maladies respiratoires du centre hospitalier universitaire Ibn Rochd de Casablanca, sur une période de 13 ans. La moyenne d’âge était de 34 ans (21-57). Un antécédent de tuberculose traitée était relevé dans un cas. Le tableau clinique était révélé par l'apparition insidieuse d'une masse pariétale de taille, de consistance et de siège variables. A l'imagerie thoracique, l'abcès pariétal était associé à une lyse osseuse dans sept cas, une atteinte parenchymateuse et pleurale dans quatre cas chacune et des adénopathies médiastinales dans deux cas. La confirmation diagnostique était bactériologique et/ou histologique dans tous les cas. La sérologie du virus de l'immunodéficience humaineétait négative chez tous nos malades. L’évolution sous traitement antibacillaire couplé ou non à une résection chirurgicale était favorable chez tous nos malades. Malgré la fréquence de la tuberculose dans notre contexte, la localisation pariétale thoracique reste rare, survenant chez une population non immunodéprimée et non toxicomane, contrairement à ce qui est souvent rapporté dans la littérature. Les abcès froids tuberculeux représentent une forme rare de tuberculose extrapulmonaire dont l’évolution reste favorable sous traitement précoce et bien conduit.

## Introduction

La tuberculose demeure une maladie d´actualité et un problème majeur de santé publique dans les pays en voie de développement. Elle peut siéger au niveau de n'importe quel organe, intéresser des localisations inhabituelles et revêtir des formes cliniques trompeuses. La localisation à la paroi thoracique est exceptionnelle et inhabituelle [1, 2]. La stratégie diagnostique et thérapeutique appropriée reste controversée vu le nombre réduits de patients rapporté dans la littérature [1–4]. L'objectif de cet article est de décrire les particularités diagnostiques, thérapeutiques et évolutives de cette entité.

## Méthodes

C'est une étude descriptive rétrospective portant sur 18 cas d'abcès froids de la paroi thoracique survenant chez des patients non infectés par le virus de l'immunodéficience humaine et diagnostiqués durant une période de 13 ans (2001 - 2013). Ils étaient exclus de cette étude, les patients ayant des abcès ossifluants du mal de Pott et les empyèmes de nécessité, de même que les abcès de la paroi thoracique sans confirmation histologique ou bactériologique.

## Résultats

**Figure 1:**
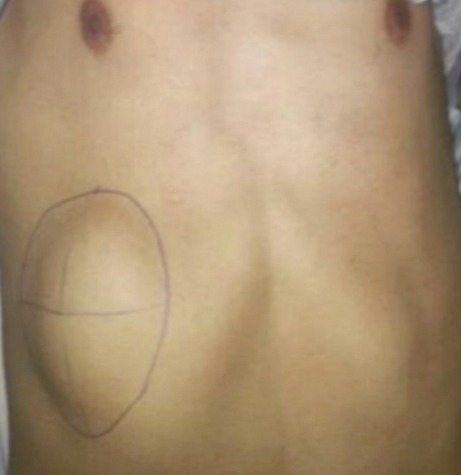
Abcès froid pariétal antérobasal droit chez un jeune homme de 26 ans, sans antécédent tuberculeux conn

Durant la période d’étude, 18 cas d'abcès froids thoraciques étaient diagnostiqués. Il s'agissait de dix femmes et huit hommes dont la moyenne d’âge était de 34 ans, avec des extrêmes allant de 21 à 57 ans. Un antécédent de tuberculose traitée était relevé dans un (5,5%) cas, par contre aucun cas de tuberculose active concomitante ni de contage tuberculeux connus n'ont été rapportés. Le tabagisme était noté dans quatre (22,2%) cas, le diabète dans deux (11,1%) cas, sans terrain de toxicomanie connu. Le début de la symptomatologie était progressif dans tous les cas. Il comportait une douleur thoracique dans la moitié des cas et l'autopalpation d´une masse pariétale thoracique (Figure 1), peu sensible dans 12 (66,7%) cas et douloureuse dans six (33,3%) cas. La fièvre était absente dans tous les cas. La taille, la consistance et le siège de la masse pariétale sont rapportés dans le Tableau 1. Les téguments en regard étaient normaux dans 15 (83,3%) cas. L'intradermoréaction à la tuberculine était positive dans tous les cas. La radiographie du thorax montrait une opacité de type pariétal dans huit (44,4%) cas, une opacité de type pleural dans quatre (22,2%) cas, des opacités excavées dans trois (16,6%) cas, une coiffe pleurale dans un (5,5%) cas et elle était normale dans trois (16,6%) cas. L´échographie thoracique, réalisée chez six (33,3%) malades, montrait une formation liquidienne à paroi épaisse dans cinq (27,7%) cas et dans un (5,5%) cas l´échostructure était tissulaire hétérogène simulant une origine tumorale.

**Tableau 1:** Tableau clinique

	Nombre	Pourcentage%
Douleur thoracique	6	33,3
Masse pariétale thoracique	18	100
**Taille: (grand diamètre, en centimètre)**		
3-1O	9	50
11-20	6	33,3
21-27	3	16,6
**Consistance**		
Ferme avec fluctuation centrale	6	33,3
Totalement fluctuante	9	50
Dure	3	16,6
Fistulisation à la peau	1	5,5
**Siège**		
Antérosupérieur	6	33,3
Antérobasal	4	22,2
Postérosupérieur	3	16,6
Postérobasal	1	5,5
Axillaire droit	3	16,6
Axillaire gauche	1	5,5

**Figure 2:**
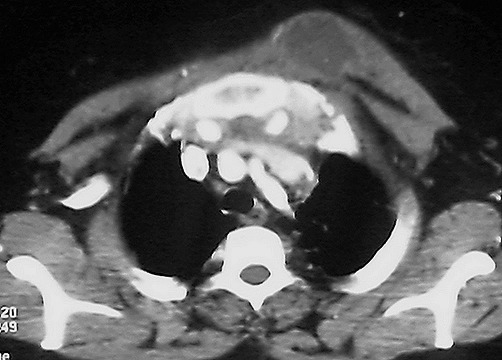
TDM thoracique montrant un abcès froid pariétal antérieur gauche

**Figure 3:**
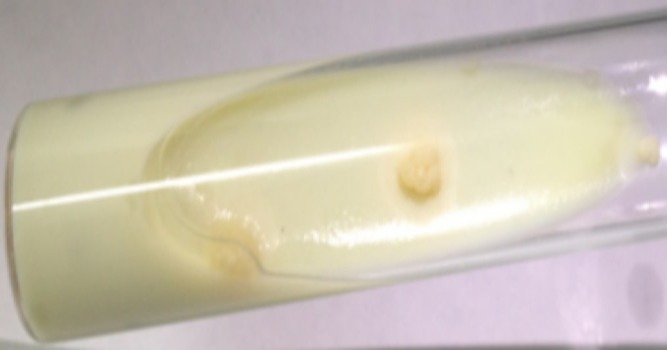
Mise en évidence duMycobacteriumtuberculosis sous forme de colonies rugueuses de couleur chamois apparaissant sous l'aspect de “verrue” ou de “chou-fleur” après culture sur le milieu de Loewenstein-Jensen

La tomodensitométrie thoracique (Figure 2), réalisée chez 11 (61,1%) malades, montrait en plus de l'abcès pariétal, une lyse costale dans quatre (22,2%) cas, sternale dans un (5,5%) cas, une lyse vertébrale et des adénopathies médiastinales dans deux (11,1%) cas chacune et une atteinte parenchymateuse et pleurale dans quatre (22,2%) cas chacune. La confirmation diagnostique était bactériologique (Figure 3) et/ou histologique dans tous les cas (Tableau 2). La sérologie du virus de l´immunodéficience humaine, réalisée chez tous les malades selon le programme national de lutte antituberculeuse, était négative. L´abcès froid pariétal était isolé dans quatre (22,2%) cas et associé à d'autres localisations tuberculeuses illustrées dans le Tableau 3. Le traitement antibacillaire était instauré dans 11 (61,1%) cas selon le régime 2SRHZ/7RH, deux mois de l'association de la Streptomycine (S), la Rifampicine (R), l'Isoniazide (I) et le Pyrazinamide (Z) suivie de sept mois de l'association RH, dans cinq (27,7%) cas le régime 2RHZE/4RH (E: Ethambutol) et 2RHZE/7RH et 2SRHZ/1RHZ/5RH dans un cas (5,5%) chacun. Une mise à plat chirurgicale de l'abcès était réalisée dans 11 (61,1%) cas et des ponctions évacuatrices dans sept (38,8%) cas. L´évolution sous traitement était favorable dans tous les cas avec disparition complète des abcès pariétaux et retour à la normale des structures osseuses érodées.

**Tableau 2:** Confirmation diagnostique

	Nombre	Pourcentage%
**Bactériologique**		
BK D du pus d'abcès	9	50
BK C du pus d'abcès	4	22,2
BK C du fragment biopsié	2	11,1
BK D dans les expectorations	2	11,1
**Histologique : Granulome épithéliogigantocellulaire**		
Avec nécrose caséeuse	8	44,4
Sans nécrose caséeuse	3	16,6

BKD: *Bacille de Koch* à l'examen direct

BKC: *Bacille de Koch* à la culture sur milieu de *Lovenstein-Jensen*

**Tableau 3:** Localisations tuberculeuses

	Nombre	Pourcentage%
Atteinte unique et isolée	4	22,2
Atteinte bifocale	11	61,1
Atteinte multiple	3	16,6
**Localisations associées**		
Pulmonaire	4	22,2
Pleurale	4	22,2
Osseuse	7	38,8
Côte	4	22,2
Vertèbre	2	11,1
Sternum	1	5,5
Ganglionnaire médiastinale	2	11,1
Ganglionnaire périphérique	1	5,5
Abcès froid inguinal	1	5,5
Abcès froid du psoas	1	5,5

## Discussion

La tuberculose demeure un problème de santé dans les pays en voie de développement et un défi de santé dans les pays développés [5–7]. La localisation pariétale thoracique en est une forme très rare. Cette définition élimine les abcès du mal de Pott, les abcès froids de la glande mammaire et aussi la propagation à la paroi d´une pleurésie purulente tuberculeuse ou «empyèmes de nécessité» [8]. Cette présentation rare et inhabituelle représente moins de 0,1% de toutes les formes de tuberculose et 1 à 5% des localisations ostéo-articulaires [1, 2, 4, 9–13]. Les atteintes costales et celles des espaces intercostaux sont les plus fréquemment rencontrées. Celles dusternum, de la clavicule, sous-costales et des parties molles sans atteinte osseuse associée demeurent exceptionnelles [1, 12–15]. La pathogénie de cette entité reste controversée. Elle est exceptionnellement primitive. Il s'agit très souvent d'une localisation survenant au cours d'une tuberculose grave et disséminée [1]. La dissémination hématogène, lymphatique ou par contiguïté est évoquée [1, 2, 4, 16–21]. En milieu agricole, l´éventualité d´une inoculation directe transcutanée au niveau de la paroi thoracique est envisagée. Un cas compliquant une vaccination par le Bacille de Calmette et Guérin (BCG) a été rapporté [19]. Le siège parasternal est la localisation préférentielle, par le biais des ganglions intercostaux antérieurs qui sont le plus souvent concernés. Dans notre série, l'abcès froid pariétal thoracique était unique et isolé dans quatre cas, et associé à d'autres localisations tuberculeuses non graves et non disséminées dans les autres cas. Ceci étant sans doute en rapport avec l'amélioration de la prise en charge des cas de tuberculose dans notre pays grâce à la mise en place du programme national de lutte antituberculeuse. L'abcès tuberculeux peut souvent se fistuliser à la peau, c'est le cas d'un seul patient dans notre série, ou rarement donner une seconde localisation [15, 19]. Il s´observe dans les deux sexes avec une légère prédominance masculine [7]. Le sexe ratio (Homme/Femme) est de 1,47 dans la série de Tsagouli et al. [22] et de deux dans la série de Aghajanzadeh et al. [17]. Par contre dans la notre, il est de 0,8. La prévalence est plus élevée chez l´adulte jeune, entre 15 et 35 ans, et exceptionnelle chez l´enfant, même en zone de forte endémie tuberculeuse[7, 16, 23]. Elle peut survenir aussi chez le sujet âgé de plus de 50 ans souvent porteur de multiples comorbidités, terrain propice pour le développement de cette affection [9, 16]. Dans notre série, la moyenne d’âge de nos patients était de 34 ans, ce qui rejoignait celle de la grande série de Paik et al. [18] et qui était de 33,3 ans. Les africains, les habitants du sous-continent indien et des Antilles sont les plus fréquemment touchés. Durant ces dix dernières années, une recrudescence de cette forme de tuberculose, notamment dans les pays en voie de développement, est rapportée [23]. Elle est plus fréquente dans la population toxicomane et chez les immunodéprimés où elle s´associe volontiers à d´autres localisations [1, 20]. Dans la présente étude, seulement 18 cas d'abcès froid thoracique sont rapportés dans notre structure, sur une période de 13 ans, chez une population non toxicomane et non immunodéprimée. Ce qui ne rejoint pas les données de la littérature, sans doute en rapport également avec l'amélioration de la prise en charge de la tuberculose dans notre contexte.

Les antécédents de tuberculose sont rencontrés chez 83% des patients et une tuberculose active est concomitante dans 17,4% à 62,5% des cas [4, 7, 9, 15, 19, 21, 24]. Dans la série récente de Keum et al. [3], 32,4% des patients avaient un antécédent de tuberculose ou une tuberculose active contre 62,9% dans celle de Paik et al. [18] et seulement 5% des cas dans la notre. Un cas d´abcès pariétal thoracique a été rapporté dans la littérature chez un homme de 80 ans ayant un antécédent d'une thoracoplastie supérieure droite pour tuberculose pulmonaire 58 ans plus tôt [24]. Sur le plan clinique, la tuberculose pariétale a une longue évolution à bas bruit[9, 13, 10, 23] et des aspects variés et trompeurs, ce qui peut être source de retard diagnostique faisant alors errer ce dernier vers celui d'abcès à pyogènes, de tumeur bénigne ou maligne [11, 12, 14, 15, 20, 22]. La taille est variable ainsi que la consistance. Ces lésions sont généralement solitaires, mais chez certains patients, de multiples lésions ont été trouvées dans deux ou plusieurs sites thoraciques ou extra thoraciques [7, 12, 17, 21]. Keum et al. [3] ont rapporté 60 cas à lésion unique sur 64, contre 86 sur 89 cas colligés par Paik et al. [18] et cinq des six cas par Kuzucua et al. [24] et chez qui le sixième avait trois abcès. Dans notre série, on a relevé deux cas chez qui l'abcès froid thoracique était associé à un abcès inguinal et du psoas dans un cas chacun. L'imagerie ne rapporte pas de signes radiologiques spécifiques [9, 10, 19]. L´échographie peut montrer une image hypoéchogène hétérogène témoignant du caractère ramolli de la masse et permettant de guider la biopsie [9, 10]. La tomodensitométrie thoracique, plus performante et plus sensible que la radiographie conventionnelle, met en évidence une masse de densité hétérogène avec des zones hypodenses centrales de nécrose avec parfois des calcifications, une destruction osseuse ou costale. Enfin, elle permet de guider la biopsie ou le drainage,faire un bilan lésionnel en recherchant d'autres localisations tuberculeuses qu'elles soient pulmonaires, pleurales sous-jacentes ou autres [4, 13, 19, 22]. Dans notre série, réalisée chez 61,1% des patients, la tomodensitométrie thoracique a permis de mettre en évidence une lyse costale, sternaleet vertébrale, des adénopathies médiastinales et une atteinte parenchymateuse et pleurale. La scintigraphie osseuse est plus performante pour dépister des localisations osseuses muettes cliniquement, voire radiologiquement, en montrant des foyers d'hyperfixation. L'imagerie par résonance magnétique montre des anomalies morphologiques et de signal de l'os et des parties molles en hyposignal T1 et intenses en T2 [3, 20]. Le diagnostic de tuberculose pariétale reste difficile en l'absence d'autres localisations pulmonaires ou extra-pulmonaires évocatrices de tuberculose, d'autant plus que d'autres affections néoplasiques ou infectieuses peuvent avoir le même aspect clinique et radiologique [11, 19, 25, 26]. De ce fait, une confirmation diagnostique bactériologique et/ou histologique s'impose. Le Mycobacterium tuberculosis pourrait être isolé dans le liquide de ponction et/ou dans les fragments de biopsiesà l'examen direct et à la culture sur milieu de Lowenstein-Jensen.

Actuellement, la polymerase chain reaction(PCR) est d'un grand apport, elle permet de poser le diagnostic précocement surtout après échec des méthodes bactériologiques classiques [3, 13, 15, 19]. L’étude histologique des biopsies des berges de l´abcès ou des pièces d'exérèses chirurgicalesde la masse pariétalepermet également d'affirmer le diagnostic en montrant une inflammation granulomateuse tuberculoïde avec nécrose caséeuse [19, 24]. Dans leur étude de 13 cas, Sakuraba et al. [3] ont rapporté des bacilles tuberculeux positifs dans neuf cas, une PCRpositive dans quatre cas et six cas avaient des cultures positives des fragments biopsiés. Dans notre série, la confirmation était bactériologique et/ou histologique de l'abcès pariétal dans tous les cas, mais également par la positivité de la recherche des bacilles de Koch à l'examen direct dans les expectorations dans deux cas. En l´absence de traitement, l´évolution peut se faire vers la fistulisation, la dissémination locorégionale et à distance. Cependant, le traitement optimal de l'abcès froid est controversé [11, 17, 24]. Certains auteurs optent pour une polychimiothérapie tuberculeuse seule [21], d'autres préconisent l'association de la chirurgie au traitement antituberculeux afin de réduire les récidives. Selon certains auteurs, cette association constitue la seule garantie de guérison définitive [3, 11, 17, 22, 24, 25]. La polychimiothérapie antituberculeuse est d'une durée de six à neuf mois en fonction de la présence ou non d´autres localisations tuberculeuses associées [10, 19, 20]. Le geste chirurgical consiste à réséquer l'abcès en totalité, emporter les tissus nécrosés sous-jacents (côtes, cartilage, sternum, adénopathies), supprimer un éventuel trajet fistuleux productif chronique et enfin si nécessaire la couverture en utilisant un lambeau musculaire [19]. Cependant, la chirurgie appropriée selon l´étendue de la lésion reste non consensuelle et des rechutes ont été rapportées au cours des dernières décennies [4]. Dans notre étude, associé au traitement antibacillaire dans tous les cas, la mise à plat chirurgicale de l'abcès était réalisée dans 61,1% des cas et les ponctions évacuatrices dans 38,8% des cas. Aucun cas de rechute n'a été enregistré.

## Conclusion

La tuberculose pariétale thoracique est une entité rare d’évolution progressive même dans un pays de forte endémie tuberculeuse. Sa présentation inhabituelle et souvent trompeuse pose souvent un problème de diagnostic nécessitant une preuve bactériologique et au mieux histologique. Cependant, sa survenue dans un contexte de toxicomanie, d'immunodépression ou de tuberculose grave n'a pas été relevée dans notre série. Le pronostic est habituellement bon sous chirurgie couplée à la chimiothérapie antituberculeuse. Le meilleur traitement passe par la prévention.

## References

[1] Gaude GS, Reyas AK (2008). Tuberculosis of the chest wall without pulmonary involvement. Lung India..

[2] Grover SB, Jain M, Dumeer S, Sirari N, Bansal M, Badgujar D (2011). Chest wall tuberculosis - A clinical and imaging experience. Indian J Radiol Imaging..

[3] Keum DY, Kim JB, Park CK (2012). Surgical treatment of a tuberculous abscess of the chest wall. Korean J ThoracCardiovasc Surg..

[4] Tanaka S, Aoki M, Nakanishi T, Otake Y, Matsumoto M, Sakurai T (2012). Retrospective case series analysing the clinical data and treatment options of patients with atubercular abscess of the chest wall. Interact CardiovascThorac Surg..

[5] Prapruttam D, Hedgire SS, Mani SE, Chandramohan A, Shyamkumar NK, Harisinghani M (2014). Tuberculosis-the great mimicker. Semin Ultrasound CT MR..

[6] Ekingen G, Guvenc BH, Kahraman H (2006). Multifocal tuberculosis of the chest wall without pulmonary involvement. Acta ChirBelg..

[7] Faure E, Souilamas R, Riquet M, Chehab A, Le Pimpec-Barthes F, Manac'h D (1998). Cold abscess of the chest wall: A surgical entity?. Ann ThoracSurg..

[8] Trombati N, Afif H, El Farouki Z, Bahlaoui A, Aichane A, Bouayad Z (2001). La tuberculose pariétale thoracique en dehors de l'immunodépression par le virus de l'immunodéficience humaine. Rev Mal Respir..

[9] El Barni R, Lahkim M, Achour A (2013). Abcès tuberculeux de la paroi thoracique chez l'enfant. Pan Afr Med J..

[10] Mahouachi R, Zendah I, Taktak S, Chtourou A, Ben Chaabane R, Gharbi R (2006). Tuberculose de la paroithoracique. Rev PneumolClin..

[11] Gayathri Devi DR, Narayanaswamy YV, Areena H (2012). Recurrent cold abscess of the chest wall in a young immunocompetent person. IJRRMS..

[12] Mosharraf H, AbulKalam A, Samprity I, Mohibul A (2010). Multiple chest wall tuberculous abscesses. J Pak Med Assoc..

[13] Madeo J, Patel R, Gebre W, Ahmed S (2013). Tuberculous Empyema Presenting as a Persistent Chest Wall Mass: Case Report. GERMS..

[14] Benyahya E, Etaouil N, Bennis R, Mkinsi O (2002). Une masse de la paroi thoracique. About a chest wall mass. Rev Med Interne..

[15] Ka AS, Brousse V, Diakhaté I, Sermet-Gaudelus I, Lenoir G, Imbert P (2006). Abcès froid tuberculeux de la paroi thoracique chez l'enfant: à propos de 3 cas. Arch Pediatr..

[16] Bekci TT, Tezcan B, Yasar S, Kesli R, Maden E (2010). Tuberculousabscess of the chestwall. Eur J Gen Med..

[17] Aghajanzadeh M, Pourrasouli Z, Aghajanzadeh G, Massahnia S (2010). Surgical treatment of chest wall tuberculosis. Tanaffos..

[18] Paik HC, Chung KY, Kang JH, Maeng DH (2002). Surgical treatment of tuberculous cold abscess of the chest wall. Yonsei Med J..

[19] Abid M, Ben Amar M, Abdenadher M, Haj Kacem A, Mzali R, Beyrouti MI (2010). Abcès de la paroi thoracique et abdominale isolé: une forme exceptionnelle de tuberculose. Rev Mal Respir..

[20] El Kharras A, Jidal M, Achemlal L, Atmane M, Chaouir S, Amil T (2004). Tuberculose sternale isolée: deux observations. Presse Med..

[21] Cho KD, Cho DG, Jo SM, Ahn IM, Park CB (2006). Current surgical therapy for patients with tuberculous abscess of the chest wall. Ann Thorac Surg..

[22] Tsagouli P, Sotiropoulou E, Filippousis P, Sidiropoulou N, Georgiadi V, Thanos L (2012). Contribution of computed tomography guided percutaneous drainage of tuberculous cold abscesses adjunctive to pharmaceutical anti-tubercular treatment. Eur J Radiol..

[23] Chermiti Ben Abdallah F, Boudaya MS, Chtourou A, Taktak S, Mahouachi R, Ayadi A (2013). Tuberculosesternale avec fracture spontanée du sternum. Rev Pneumolclin..

[24] Kuzucua A, Soysala O, Gunenb H (2004). The role of surgery in chest wall tuberculosis. Interact CardiovascThorac Surg..

[25] RivoVázquez JE, Fernández Villar A, CañizaresCarreteroa MA (2004). Cold abscess of the chest wall 58 years after thoracoplasty. Arch Bronconeumol..

[26] Kamiyoshihara M, Ibe T, Iwasaki Y, Takise A, Itou H, Takeyoshi I (2010). Chest wall tuberculoma with tumor-like appearance. Respir Med CME..

